# Guillain-Barrè Syndrome—Retrospective Analysis of Data from a Cohort of Patients Referred to a Tertiary Care Pediatric Neuromuscular Center from 2000 to 2017: Electrophysiological Findings, Outcomes, and a Brief Literature Review

**DOI:** 10.3390/medicina60091490

**Published:** 2024-09-12

**Authors:** Benedetta Cavirani, Margherita Baga, Carlo Alberto Cesaroni, Susanna Rizzi, Carlotta Spagnoli, Daniele Frattini, Elvio Della Giustina, Francesco Pisani, Carlo Fusco

**Affiliations:** 1 Child Neurology and Psychiatry Unit, Department of Pediatrics, AUSL-IRCCS di Reggio Emilia, 42121 Reggio Emilia, Italy; benedetta.cavirani@gmail.com (B.C.); magia1689@gmail.com (M.B.); susanna.rizzi@ausl.re.it (S.R.); carlotta.spagnoli@ausl.re.it (C.S.); daniele.frattini@ausl.re.it (D.F.); elvio.dellagiustina@gmail.com (E.D.G.); carlo.fusco@ausl.re.it (C.F.); 2 Child Neuropsychiatry Unit, Azienda USL di Parma, 43121 Parma, Italy; 3 Child Neurology and Psychiatry Unit, Department of Human Neuroscience, Sapienza University, Via dei Sabelli 108, 00185 Rome, Italy; francesco.pisani@uniroma1.it

**Keywords:** Guillain-Barré, outcome, pediatric age, treatment

## Abstract

*Background and Objectives:* Guillain-Barré syndrome (GBS) is the most frequent cause of acute flaccid paresis in children. The aim of this study was to describe the clinical and electrophysiological findings and outcomes of children with GBS diagnosed in our unit. Moreover, the literature on pediatric GBS cases from the past 5 years was reviewed. In this retrospective study, we reported data on 12 patients (9 male and 3 female patients; mean age: 5 y, 4 mo; range: 9 mo–11 y) clinically diagnosed at the Child Neurology Unit of the AUSL-IRCCS of Reggio Emilia, Italy, between 2000 and 2017 and a brief analysis/comparison with data from the literature. *Materials and Methods:* Data were collected from medical charts. *Results:* In our cohort, male patients were more frequent than female ones (9 vs. 3), and upper respiratory tract infection (n = 8, 66.7%) was the most frequent triggering factor. The main clinical symptoms on admission were distal lower limbs’ weakness with gait difficulties (83.3%), pain (50%), upper limbs’ weakness (50%), and dysphagia for liquids (25%). Peripheral neurophysiological studies revealed acute inflammatory demyelinating polyradiculoneuropathy (AIDP) in 66.6% of the children, acute motor and sensory axonal neuropathy (AMSAN) in 25%, and acute motor axonal neuropathy (AMAN) in 8.3%. Ten individuals (83.3%) received timely treatment with intravenous immunoglobulins (IVIG), and, out of these ten patients, 58% received concomitant treatment with IV methylprednisolone because of a progressive disease course. Complete remission was observed in the majority of individuals (91.6%) within 6 months of symptom onset. *Conclusions:* Different subtypes of GBS can affect children; however, the outcome is usually positive. Early treatment appears to be important for a favorable outcome.

## 1. Introduction

Guillain-Barré syndrome (GBS) is the most frequent cause of acute flaccid paresis in the pediatric population [1]. It is characterized by the rapid onset of bilateral and symmetric sensorimotor signs and symptoms, progressively involving the lower limbs and upper limbs. The clinical presentation can be heterogeneous; in more severe cases, the autonomic nervous system can also be impaired. Frequently, trivial infections (i.e., an upper respiratory tract or gastrointestinal infection) before the onset of neurological symptoms can act as a trigger [1]. Usually, this disease is prevalent in males [2]. There are several clinical subtypes of GBS that differ in terms of clinical and neurophysiological features (i.e., demyelinating and axonal forms) and long-term outcomes. In particular, electrophysiological studies help distinguish among the various subtypes of GBS, namely (a) the acute inflammatory demyelinating polyneuropathy (AIDP), which is the most frequent type in North America and Europe [3], (b) the acute motor axonal neuropathy (AMAN), and (c) the acute motor–sensory axonal neuropathy (AMSAN). Rarer subtypes are characterized by an atypical clinical presentation (such as Miller Fisher syndrome, the pharyngo-cervical-brachial variant, and Bickerstaff encephalitis). We report hereunder our retrospective experience in children with GBS, describing their clinical and electrophysiological findings and outcomes, alongside a review of the literature on pediatric patients with GBS from the past 5 years.

## 2. Results

We hereby report the cases of 12 individuals with GBS. All children consecutively admitted to the Child Neurology Unit with GBS between 2000 and 2017 were enrolled in the present retrospective study (Table 1). For each patient the following variables were collected: gender, age at the onset of clinical presentation, antecedent events, seasonal distribution, neurological signs and symptoms, electrophysiological findings, laboratory investigations (i.e., cerebrospinal fluid examination results, serum antiganglioside antibody positivity), imaging features, treatment, and clinical course. After ethical committee approval, data were collected from the patients’ medical charts. Parental written consent was obtained for each individual. After discharge from the hospital, the patients underwent follow-up at the same Child Neurology Unit. Electrophysiological evaluations were performed during the early hospitalization period, two weeks later, one and six months later, and, afterwards, according to clinical needs. Motor and sensor nerve conduction velocities and F-wave responses were recorded by surface electrodes upon supra-maximal percutaneous stimulation. Usually, measurements of conduction velocities and F-wave responses were carried out on the median, ulnar, tibial, peroneal, and sural nerves of both sides. Further nerves were studied if needed. For all individuals, strength was assessed with the Medical Research Council (MRC) scale, while their functional outcomes were determined with the GBS Score, the Modified Rankin scale, and the GBS Disability Scale. Assessments were performed at the time of onset, at the peak, and during the recovery phases of the disease [4]. Moreover, the literature on pediatric patients with GBS was reviewed. The search was limited to articles in English, and it was performed on the PubMed database using the Boolean expressions (Guillain-Barré syndrome) AND (children) OR (pediatric). Because of the important and fast research development in this field, we limited our literature review to manuscripts published in the last five years. Review, letters, and commentaries were excluded from our research. Nine patients were males, and three were females. The mean age of the individuals at the time of symptom onset was 5 years and 4 months [range 9 months–11 years]. Regarding the relationship between seasonal distribution and the incidence of GBS, winter was the season with the highest rate of cases (five patients). Nine children (75%) presented antecedent events: an upper respiratory tract infection—i.e., an URTI—in four patients, chicken pox in two, and one patient each with a gastrointestinal infection, CMV infection, and dermatitis with erythema. On admission, the main clinical features were distal lower limbs’ weakness with gait difficulties or inability to walk in 83.3%, pain (located in the back or lower limbs) in 50% of the cases, upper limbs’ weakness in 50%, and dysphagia for liquids in 25%. Sensory symptoms (i.e., paresthesia or sensory loss) were also reported (one patient). In most patients, the physical examination on admission revealed impaired reflexes (nine patients presented lower limb areflexia with upper limb hyporeflexia). In four patients, we observed an ataxic gait with instability. In one patient only, cranial nerve involvement (i.e., peripheral facial nerve palsy) was present [5]. None of the patients in our sample presented with autonomic involvement or respiratory problems. Cerebrospinal fluid (CSF) analysis was performed in four patients, and albuminocytological dissociation was evident in three. Eight children had no CSF study performed on them. Serum antiganglioside autoantibodies were tested in eight patients, and, among them, four were positive. Antibodies were most frequently directed against GM1 and GM2 gangliosides. Peripheral neurophysiological studies were performed in all patients during the first day of hospitalization and revealed the following: acute demyelinating neuropathy (AIDP) in eight patients; acute motor and sensory axonal neuropathy (AMSAN) in three patients; and acute motor axonal neuropathy (AMAN) in one child. The electrophysiological findings of the patients with AIDP were reduced conduction velocities, reduced sensory- and motor-evoked amplitudes, prolonged distal motor latency, prolonged or absent F-wave, and increased temporal dispersion. Axonal GBS was characterized by decreased motor amplitudes and the absence of demyelinating features. One of the patients with AMSAN presented with erythema in the face and extremities followed by muscle weakness one month later. Furthermore, the presence of high levels of CK (creatine kinase) and myopathic EMG (electromyography) findings led to the diagnosis of an associated dermatomyositis. Nine patients underwent neuroimaging (i.e., brain and spine magnetic resonance imaging, MRI), with the enhancement of spinal nerve roots and the cauda equina in only one patient. In the other cases, imaging was normal, except for one patient showing a vascular malformation in the left hemisphere (incidental finding). Ten patients received timely treatment (within 2 weeks of weakness onset) with intravenous immunoglobulins (IVIG) (0.4 g/kg daily for 5 consecutive days) [6]. Among these, seven were treated with IVIG and intravenous methylprednisolone followed by oral prednisone for three weeks, two patients (16.6%) were treated only with IVIG, and one was treated with IVIG in association with acyclovir (because of concomitant chicken pox). Only one patient underwent a second IVIG cycle because of disease relapse. At the 6-month follow-up, complete clinical and neurophysiological recovery was present in eleven patients (91.6%), while, in one case with AIDP, a mild left leg paresis was still present.

## 3. Discussion and Conclusions

Most past pediatric studies report that the incidence of GBS is higher in males [1,7,8], with variable seasonal incidence, as some report a peak in the summer [9,10] and others in the cold seasons (autumn or winter) [11]. No difference in seasonal distribution has yet been described between the various GBS subtypes. In the literature, the most commonly reported antecedent event is an URTI, followed by a gastrointestinal infection [12,13]. The main clinical feature at onset is an ascending paralysis characterized by muscle weakness [7,8,9]. In the recent literature, dysphagia was only reported in one case of axonal GBS [14]. The frequency of cranial nerve involvement, ranging from Bell’s palsy to bulbar involvement and ophthalmoplegia, is variable between the considered studies, ranging from about 50% [3] to only a few cases [7,8]. Neuropathic pain post hospital admission was frequently reported in previous studies [3,14,15,16]. In our study, the time between the onset of symptoms and the NCV(Nerve Conduction Velocity) study, which was always pathological, averaged 7 days. By symptom onset, we mean the onset of nearly disabling motor symptoms, with ataxia, walking disorder, and the involvement of cranial nerves with motor relevance. Painful symptomatology alone, in the absence of motor signs, was not considered to signify symptom onset. Also worth noting is the lack of significant differences between the different types of Guillain-Barré syndrome described; in particular, in our patients, the type (AMAN, AIDP, AMSAN) did not affect the more or less rapid positivization of the NCV examination with respect to the onset of symptoms. Regarding treatment, the majority of studies relied on intravenous immunoglobulin (IVIG), while the combined use of steroids and IVIG was reported only in three papers and in subjects with severe symptoms [10,14,17]. However, we also found authors who did not administer any specific treatment (i.e., IVIG or plasma exchange) in addition to supportive care [16]. Furthermore, the initial disease severity appears to be similar between the different subtypes of GBS, while the long-term outcomes show some differences, being worst in those patients with more specific axonal involvement [3,14]. In our study, we described a retrospective, single-center cohort of 12 pediatric patients with GBS, comparing their clinical and neurophysiological features with those reported in the most recent literature. In accordance with previous studies, we found a higher incidence of GBS in males [1,7,8]. An URTI was the most common infectious antecedent, as previously reported in [4,9]. The most frequent signs observed at onset were abnormal deep tendon reflexes at the lower limbs (9/12), hyposthenia (12/12), and neuropathic pain. Only one child showed peripheral facial nerve palsy, despite GBS cranial nerve involvement usually being more frequent in children than in adults, especially facial palsy [7]. The abovementioned patient presented with a CMV-associated axonal sensory motor GBS (already reported in [5]). Several past studies mentioned neuropathic pain, especially to the lower limbs, as a frequent clinical manifestation in the acute phase [1,15]. This was confirmed by our patients, who reported pain in their legs and back, responsive to paracetamol. In our cohort, dysphagia for liquids was observed in a quarter of the patients, but this feature is not frequently reported in the literature. None of our children had signs of autonomic dysfunction (i.e., tachycardia, hypertension or hypotension, urinary retention), and the patients most severely affected presented a GBS disability score of ≥3 on admission. This onset score was similar in both AIDP and AMAN/ASMAN subtypes. In the literature, the correlation between dermatomyositis and peripheral neuropathy, both sensory and motor [18,19,20,21], is not new. In our case, however, neuropathy appeared not to be correlated with a chronic neuropathic phenomenon, but rather be more attributable to an acute form of neuropathy, compatible with the Guillain-Barré syndrome. The acute onset of the symptomatology, with difficulty in walking, and the fact that the NCV examination and clinical data normalized after therapy with a sufficient infusion of immunoglobulins and the use of methylprednisolone IV and methotrexate hint at a neuropathy that does not have a chronic basis but an acute basis instead, that is completely resolved.

Although erythema is quite a rare feature of GBS, we observed it in one child. Previously, only two patients with an episodic and intermittent erythema related to their autonomic dysfunction [22,23] had been described. A GBS diagnosis is based on a combination of clinical and paraclinical findings, including an increased protein level with a normal cell count in the cerebrospinal fluid (CSF) [4]. In our cohort, a CSF analysis was performed in four patients, and albuminocytological dissociation was observed in three of them (25%). A lumbar puncture with subsequent CSF protein analysis and cell count measurements comprise an ancillary investigation for GBS diagnosis. Normal CSF protein levels in the first two weeks after disease onset are mainly useful in order to rule out alternative diagnoses but not to exclude a GBS diagnosis [2]. Therefore, for diagnostic certainty in the event of initially normal CSF counts and protein concentrations, a lumbar puncture may be repeated several days (10–14 days) after symptom onset. However, the repetition of the CSF analysis can be suggested only when other findings remain inconclusive. The recent consensus-based guideline [4] recommends basing the diagnosis on clinical criteria supported by CSF and electrophysiological findings. An electrophysiological study is essential for confirming a GBS diagnosis and identifying its different variants. In our sample, AIDP was the most frequent neurophysiological variant (n = 8, 66.6%), followed by AMSAN (n = 3, 25%), while AMAN was diagnosed only in one patient. The most important differential diagnoses in GBS are hereditary neuropathies with acute onset. In particular, an acute polyradiculoneuritis-like symptomatology was reported in Charcot–Marie–Tooth syndrome type X1 (CMT1X). CMT1X is an X-dominant inherited peripheral neuropathy due to pathogenic variants in the *GJB1* gene (Xq13.1), which encodes for connexin 32, a transmembrane protein [24,25]. This type of CMT should be suspected in patients with an acute and transient episode of neurologic dysfunction, in particular with weakness and dysarthria [21]. In our study, complete remission was observed in the majority of patients within 6 months. In one case, data about the therapy were not available. All patients with a complete recovery received timely treatment with IVIG. No one received plasmapheresis. The IVIG treatment was preferred over PE, as it is less invasive, and, in accordance with recent guidelines, the IVIG treatment was started in a timely manner when our patients showed a rapid progression of weakness and the inability to walk unaided [2,4]. Some of our patients also received IV methylprednisolone, based on a previously described positive experience [5]. The combined use of IVIG and steroids is controversial. Some studies in the literature report that corticosteroids added to IVIG [14] are more efficacious in patients with severe symptoms [26]. However, the recent consensus-based practical guidelines for the diagnosis and treatment of GBS in childhood and adolescence recommend corticosteroids for those cases with an acute onset of chronic inflammatory demyelinating polyneuropathy (A-CIDP) due to a prolonged disease course (beyond 4 weeks in children) [4]. Seven patients described in our study were treated with IV methylprednisolone because the immunoglobulin treatment had been ineffective. Indeed, while the patients treated with IV methylprednisolone (for 5 days) had not shown significant improvement after the immunoglobulin course, they showed improvement after the IV methylprednisolone course; however, it is difficult to prove whether this improvement was due to a delayed action of the immunoglobulins rather than an immediate effect of the IV methylprednisolone therapy. OS cortisone therapy (not recommended in the most recent guidelines) [27] administered at home was intended to avoid a therapeutic rebound phenomenon from the abrupt discontinuation of high-dose IV methylprednisolone. Although some studies report a more severe prognosis for axonal variants, all our patients showed a good outcome, without differences between the various subtypes [3,14]. In conclusion, the clinical presentation of our patients with GBS was similar to that reported by previous studies in the literature. Nevertheless, some symptoms complained by our patients were reported less often. One of these was neuropathic back pain, which was present in a quarter of our patients, and dysphagia, especially for liquids. Luckily, no patients needed secretion management (aspiration of saliva or intubation). To date, there are no studies pertaining to the prevalence of dysphagia in children with GBS [4]. As previously mentioned, a good outcome was seen in most of our patients, but whether this was related to the prompt administration of treatment should be further investigated.

## Figures and Tables

**Table 1:** Clinical and instrumental data of individuals with Guillain-Barré syndrome.

	Pt 1	Pt 2	Pt 3	Pt 4	Pt 5	Pt 6	Pt 7	Pt 8	Pt 9	Pt 10	Pt 11	Pt 12
**Gender**	M	M	M	M	M	M	M	F	M	F	F	M
**Age at onset**	2 y 10 m	2 y 8 m	11 y	4 y 5 m	10 y 11 m	3 y 8 m	4 y 10 m	3 y 4 m	8 y 11 m	NA	9 m	11 y
**Onset**	Acute	Acute	Acute	Acute	Acute	Acute	Acute	Acute	Acute	NA	NA	Acute
**Seasonal distribution**	Autumn	Winter	Summer	Summer	Winter	Winter	Autumn	Autumn	Winter	NA	NA	Spring
**Preceding events (weeks before onset)**	Upper respiratory tract infection (2 weeks)	Chicken pox (2 weeks) and caught and diarrhea (4 weeks)	Upper respiratory tract infection (1 week)	Chicken pox ongoing	Gastroenteritis (2 weeks)	Not reported	Upper respiratory tract infection	Upper respiratory tract (2 weeks	Febrile illness (1 week)	NA	NA	Not reported
**Clinical presentation at onset**	Abnormal gait and dysphagia with liquids	Abnormal gait; dysphagia with liquids; and irritability	Abnormal gait; myalgia; and Headache	Abnormal gait and myalgia	Abnormal gait and back pain	Abnormal gait; myalgia; and back pain	Abnormal gait	Abnormal gait and myalgia	Upper limb hyposthenia; myalgia; and facial paresthesia	NA	NA	Lower and upper limb weakness
**Neurological examination on admission**	Lower limbs: hyposthenia, areflexia, and pain Upper limbs: hyposthenia	Lower limbs: hyposthenia and areflexia	Lower limbs: areflexia and generalized hyposthenia	Lower limbs: areflexia Upper limbs: hyporeflexia	Lower limbs: areflexia Romberg sign: positive	Lower limbs: areflexia Steppage gait	Lower limbs: areflexia Ataxia	Lower and upper limbs: areflexia	Lower limbs: areflexia and hypostenia Upper limbs: hyporeflexia and hyposthenia Romberg sign: positive Ataxia Right peripheral facial nerve palsy	NA	NA	Lower and upper limbs: hyposthenia and hyporeflexia
**CSF**	Albuminocytological dissociation	NP	Albuminocytological dissociation	20 cell, PCR positive for ZVZ	Normal	Albuminocytological dissociation	NP	NP	NP	NA	NA	NP
**EMG/NCV**	AIDP	AIDP	AIDP	AIDP	AIDP	AMAN	AIDP	AIDP	AMSAN	AMSAN	AIDP	AMSAN
**Anti-GM2 Serology**	-	-	-	NA	+	NA	+	+	+	NA	NA	+
**Viral serology**	Low positive anti-CMV IgM	Low positive anti-Toxoplasma IgM	Positive anti-Enterovirus IgM and IgG	Positive anti-Enterovirus IgM	NA	Negative	Positive anti-CMV IgM and IgG	Negative	Negative	NA	NA	NA
**Spinal MRI**	NP	NP	Normal	Normal	NP	Normal	Enhancement	Normal	NP	NA	NA	NA
**Therapy**	IVIG started on day 7 and IV MP followed by P	IVIG started on day 7 and IV MP followed by P	IVIG and IV MP followed by P orally	IVIG and IV acyclovir and ceftriaxone	IVIG and IV MP followed by P	IVIG	First cycle of IVIG Second cycle of IVIG and IV MP	IVIG	IVIG and IV MP followed by deflazacort	NA	NA	IVIG and IV MP followed by deltacortene
**Outcome**	Recovery	Recovery	Recovery	Recovery	Recovery	Recovery	One relapse after the first cycle of IVIG	Recovery	Recovery	NA	Lower left limb hyposthenia	Recovery

Legend: IVIG, intravenous immunoglobulin; IV MP, intravenous methylprednisolone; P, oral prednisone; NP, not performed; NCV, Nerve Conduction Velocity and NA, not available.

## Data Availability

The data used for this paper will be made available by the corresponding author upon reasonable request.

## Abbreviations

GBS	Guillain-Barrè syndrome
AMSAN	Acute motor and sensory axonal neuropathy
AMAN	Acute motor axonal neuropathy
IVIG	Intravenous immunoglobulin
MRC	Medical Research Council
URTI	Upper respiratory tract infection
CSF	Cerebrospinal fluid
AIDP	Acute demyelinating neuropathy
EMG	Electromyography
CMT	Charcot–Marie–Tooth
CIDP	Chronic inflammatory demyelinating polyneuropathy
